# Supplementary material for: Ecological stoichiometric characteristics of soil-moss C, N, and P in restoration stages of karst rocky desertification
Source: PLoS One. 2021 Jun 30;16(6):e0252838. doi: 10.1371/journal.pone.0252838 (PMC8244914; doi:10.1371/journal.pone.0252838)
Supplement: Attachment — Submitted filename: Comments to the author.docx [file pone.0252838.s002.docx]

Comments to the author

I. General comments

The authors' manuscript " Ecological stoichiometric characteristics of soil-moss C, N, and P in restoration stages of karst rocky desertification " studied the stoichiometric characteristics of moss and soil. The authors found that soil nutrients in the shrub stage are higher than other restoration stages and the growth of some moss is not affected by the poor rocky desertification soil. Moss could be used as a supplementary method in promoting ecological restoration in areas undergoing karst rocky desertification due to their positive effects on soil nutrients. Those findings may motivate researchers to better understanding the mechanisms of moss in karst areas. It is worthy to publish this manuscript in the journal of " PLOS ONE ". However, this manuscript is not organized very well. It still needs revision.

II. Specific comments

1) Line 48,50,52 (Abstract):" Studying the stoichiometric characteristics of bryophytes and soil", please do not use two different ways to express moss in manuscript.
2) Line 64-66: Please revise the expression. The Abstract should be needs to be condensed.
3) Line 99: Please add the citations.
4) The Introduction should be further introduced some research on the stoichiometry of moss.
5) Line 228-229: Please revise the expression.
6) Line 257:” related to P. C and N had significant negative correlations”, please add appropriate punctuation marks to make sentence correct.
7) Line 287-293: Please revise the expression.
8) Line 293-294,297-298,331-332,329,340-342,364-365 and 392-394: Please add the citations.
9) Line 299-300: Please revise the expression.
10) Line 331-333Pay attention to their logical relationships.
11) Line 335-337These lines look like conclusion? Shouldn't be in the discussion?
12) The Conclusion should be needs to be condensed.
13) Figures

If you can add pictures of moss in karst rocky desertification areas, it will be more helpful to readers understand the study background.
